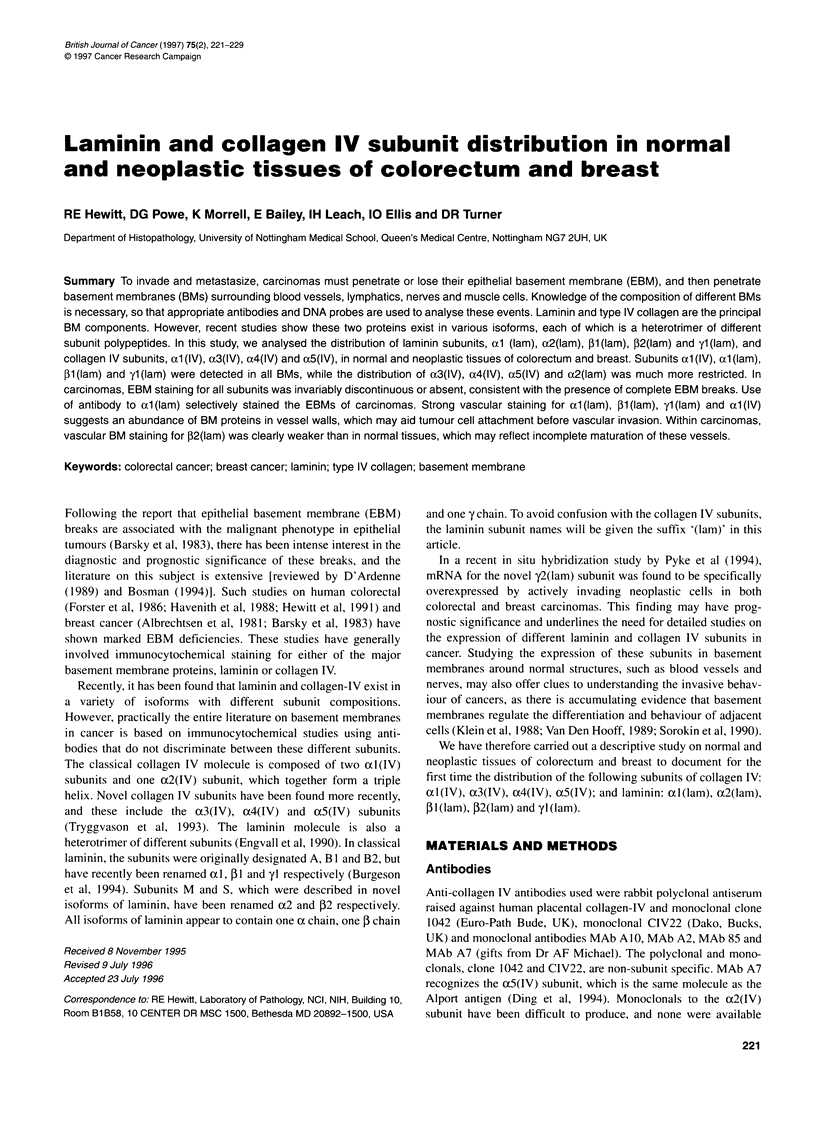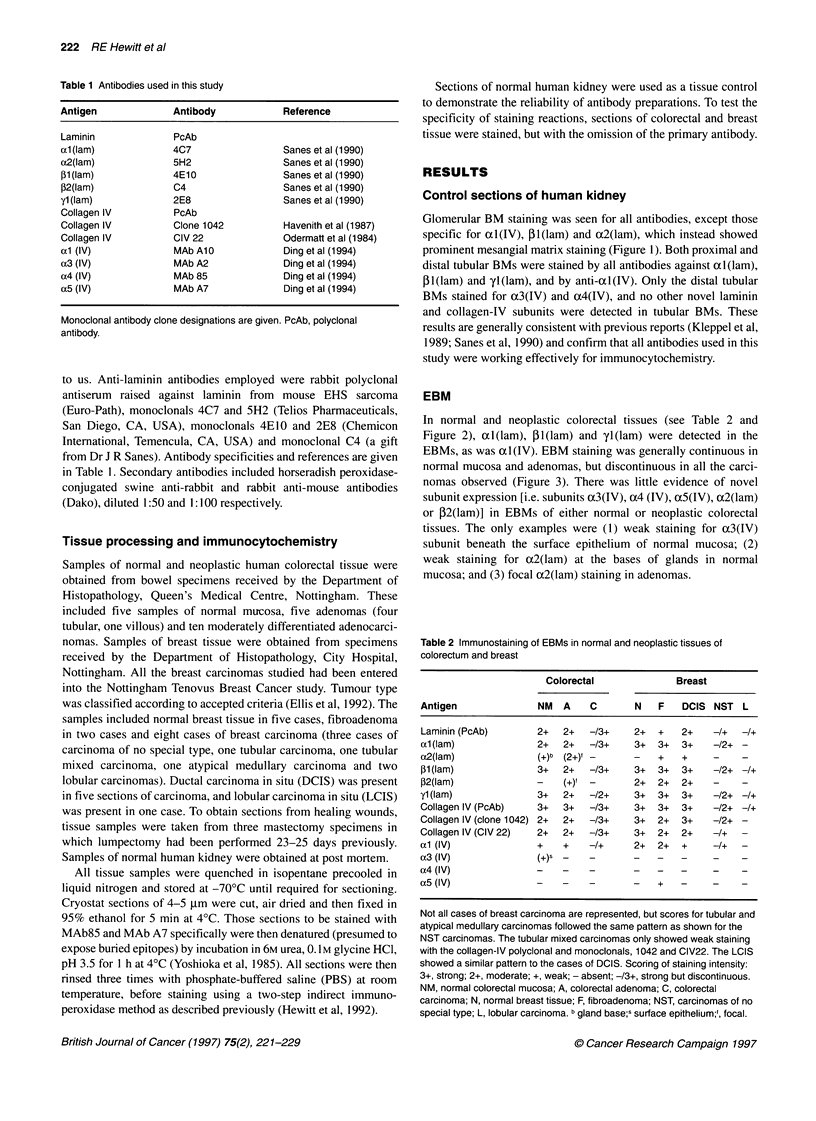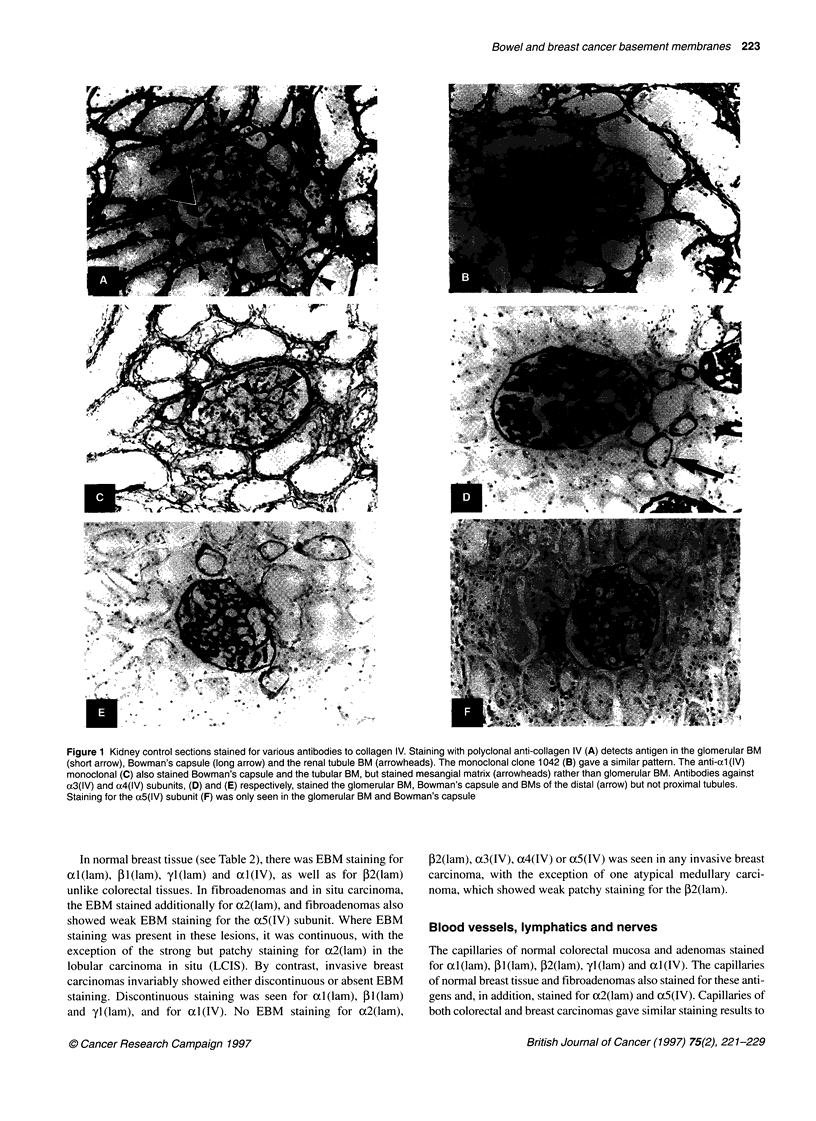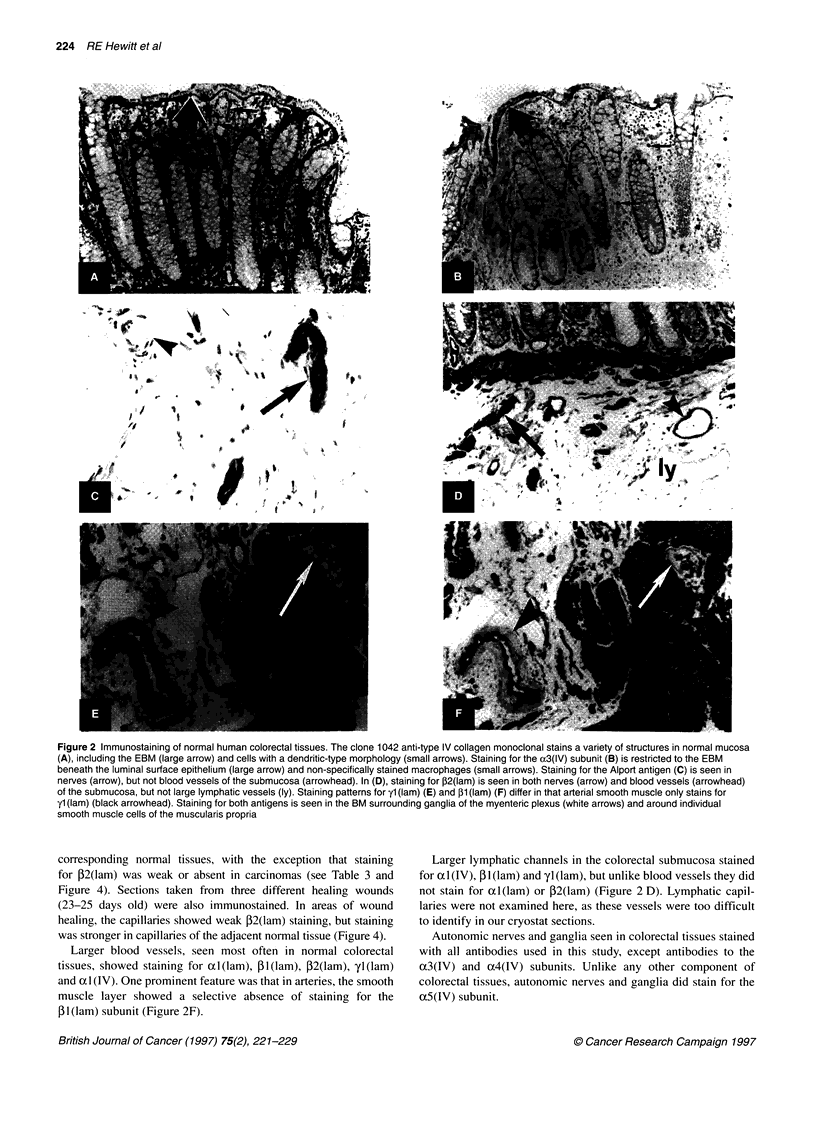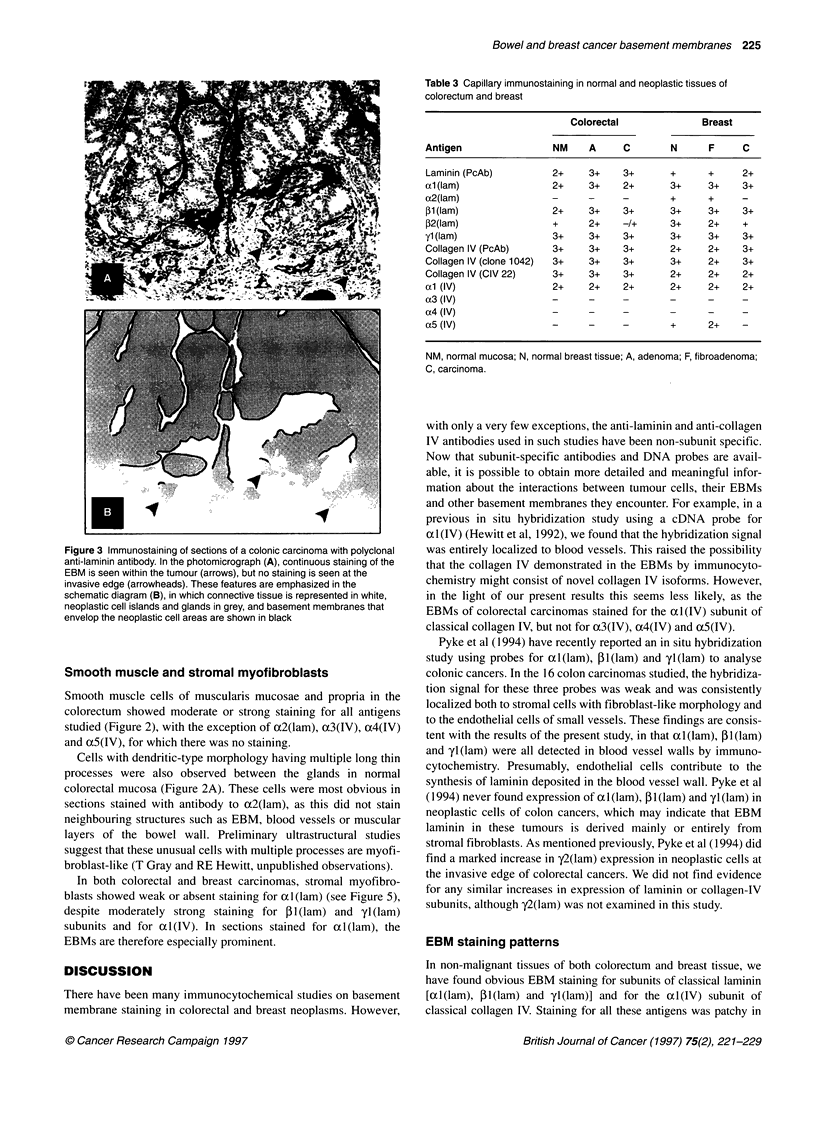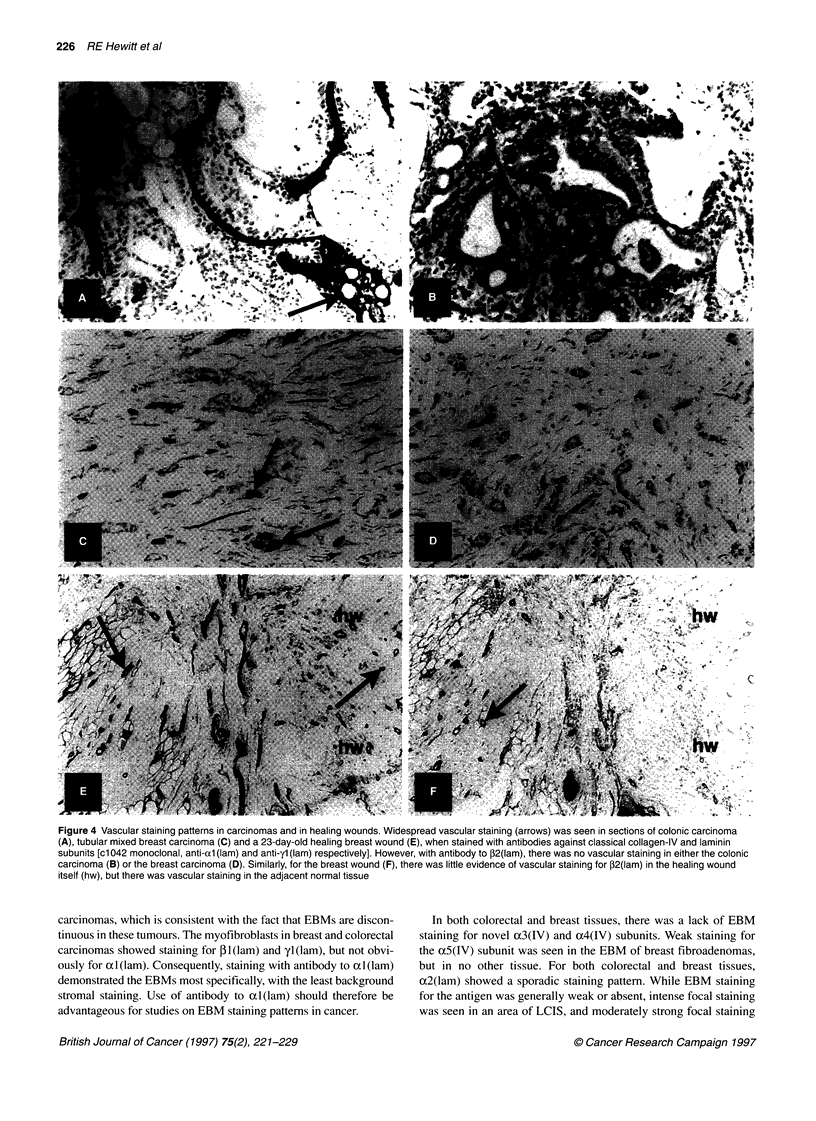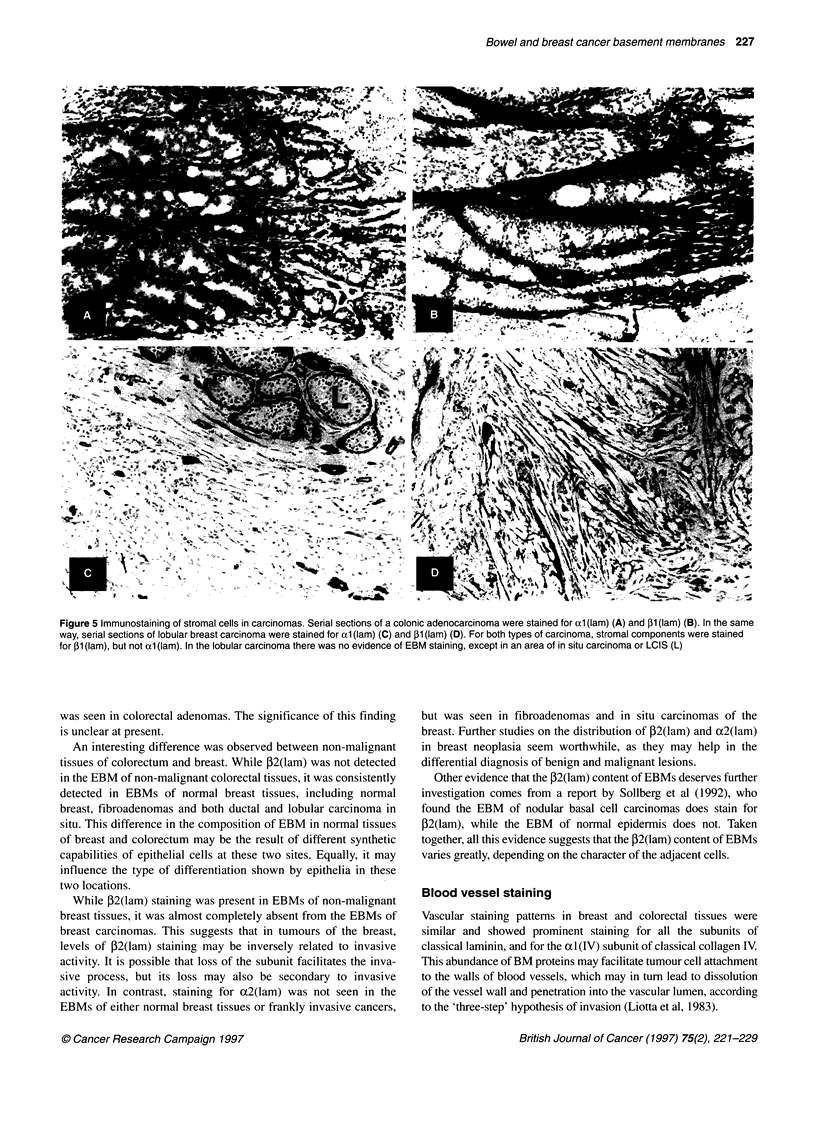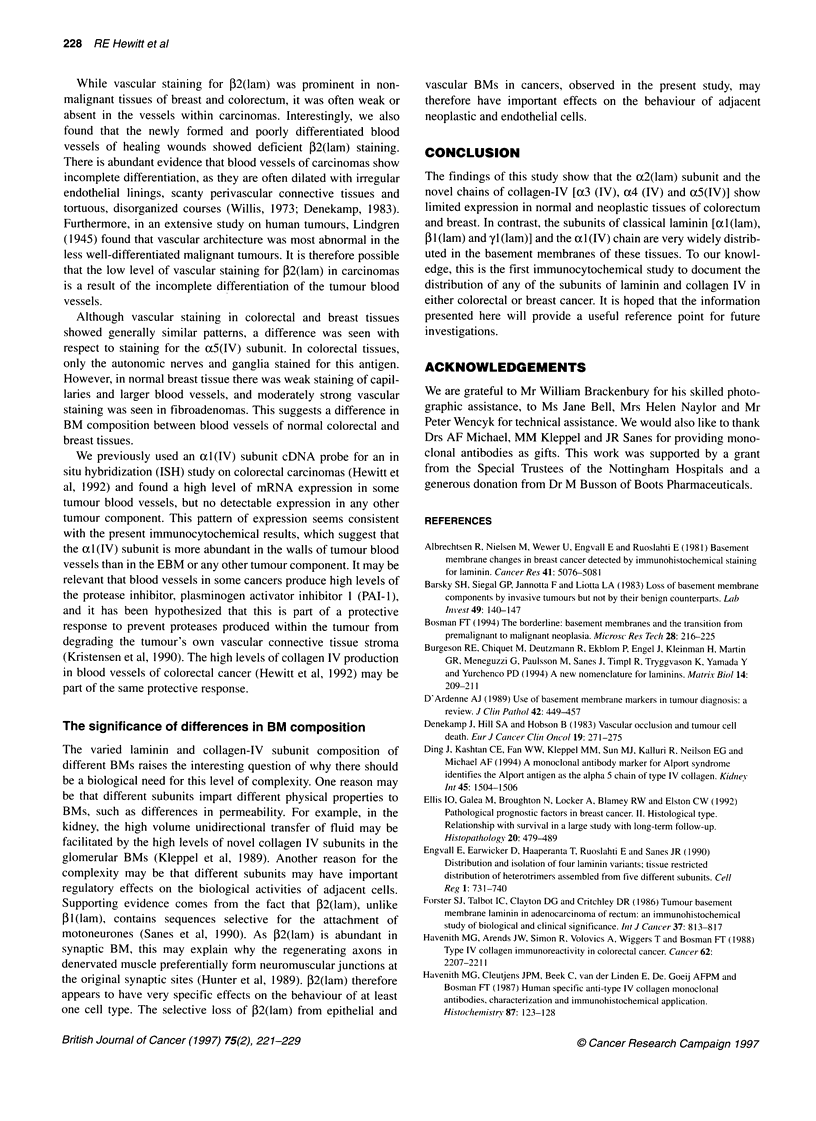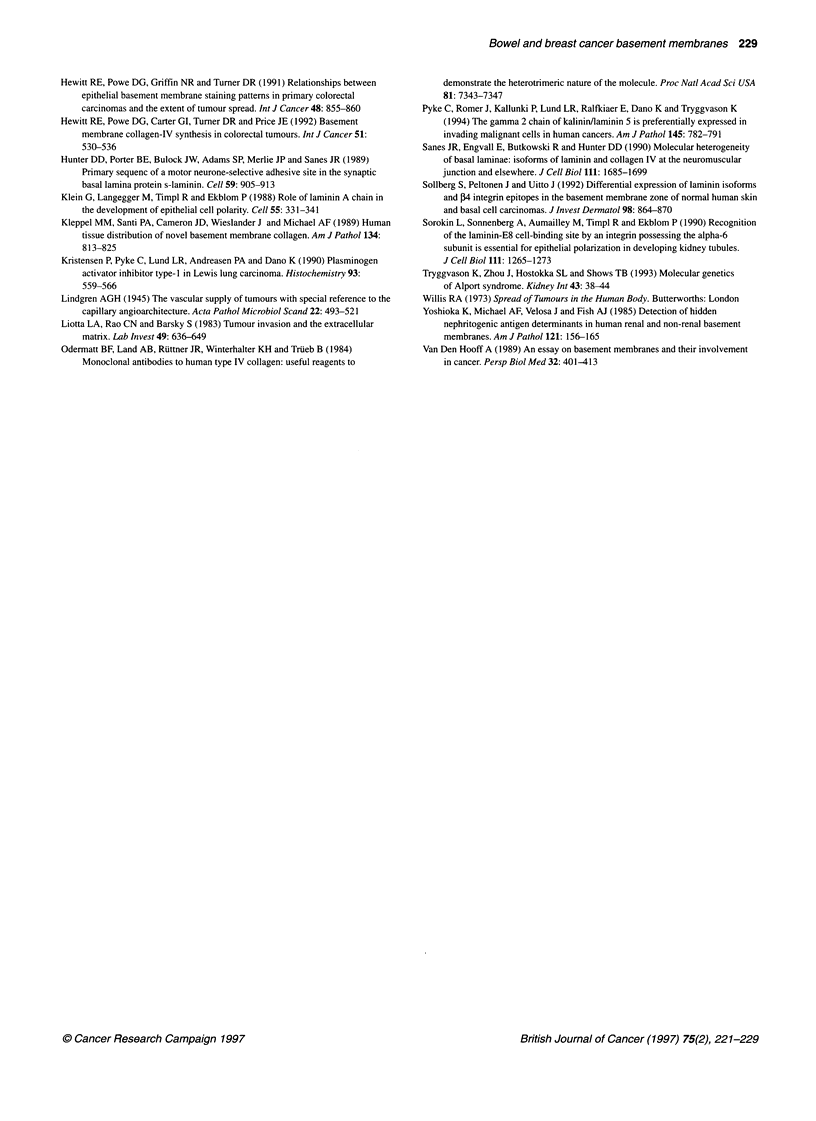# Laminin and collagen IV subunit distribution in normal and neoplastic tissues of colorectum and breast.

**DOI:** 10.1038/bjc.1997.37

**Published:** 1997

**Authors:** R. E. Hewitt, D. G. Powe, K. Morrell, E. Balley, I. H. Leach, I. O. Ellis, D. R. Turner

**Affiliations:** Department of Histopathology, University of Nottingham Medical School, Queen's Medical Centre, UK.

## Abstract

**Images:**


British Journal of Cancer (1997) 75(2), 221–229
© 1997 Cancer Research Campaign

### Laminin and collagen IV subunit distribution in normal and neoplastic tissues of colorectum and breast

**RE Hewitt, DG Powe, K Morrell, E Bailey, IH Leach, IO Ellis and DR Turner**

Department of Histopathology, University of Nottingham Medical School, Queen's Medical Centre, Nottingham NG7 2UH, UK

**Summary** To invade and metastasize, carcinomas must penetrate or lose their epithelial basement membrane (EBM), and then penetrate basement membranes (BMs) surrounding blood vessels, lymphatics, nerves and muscle cells. Knowledge of the composition of different BMs is necessary, so that appropriate antibodies and DNA probes are used to analyse these events. Laminin and type IV collagen are the principal BM components. However, recent studies show these two proteins exist in various isoforms, each of which is a heterotrimer of different subunit polypeptides. In this study, we analysed the distribution of laminin subunits, α1 (lam), α2(lam), β1(lam), β2(lam) and γ1(lam), and collagen IV subunits, α1(IV), α3(IV), α4(IV) and α5(IV), in normal and neoplastic tissues of colorectum and breast. Subunits α1(IV), α1(lam), β1(lam) and γ1(lam) were detected in all BMs, while the distribution of α3(IV), α4(IV), α5(IV) and α2(lam) was much more restricted. In carcinomas, EBM staining for all subunits was invariably discontinuous or absent, consistent with the presence of complete EBM breaks. Use of antibody to α1(lam) selectively stained the EBMs of carcinomas. Strong vascular staining for α1(lam), β1(lam), γ1(lam) and α1(IV) suggests an abundance of BM proteins in vessel walls, which may aid tumour cell attachment before vascular invasion. Within carcinomas, vascular BM staining for β2(lam) was clearly weaker than in normal tissues, which may reflect incomplete maturation of these vessels.

**Keywords:** colorectal cancer; breast cancer; laminin; type IV collagen; basement membrane

Following the report that epithelial basement membrane (EBM) breaks are associated with the malignant phenotype in epithelial tumours (Barsky et al, 1983), there has been intense interest in the diagnostic and prognostic significance of these breaks, and the literature on this subject is extensive [reviewed by D'Ardenne (1989) and Bosman (1994)]. Such studies on human colorectal (Forster et al, 1986; Havenith et al, 1988; Hewitt et al, 1991) and breast cancer (Albrechtsen et al, 1981; Barsky et al, 1983) have shown marked EBM deficiencies. These studies have generally involved immunocytochemical staining for either of the major basement membrane proteins, laminin or collagen IV.

Recently, it has been found that laminin and collagen-IV exist in a variety of isoforms with different subunit compositions. However, practically the entire literature on basement membranes in cancer is based on immunocytochemical studies using antibodies that do not discriminate between these different subunits. The classical collagen IV molecule is composed of two α1(IV) subunits and one α2(IV) subunit, which together form a triple helix. Novel collagen IV subunits have been found more recently, and these include the α3(IV), α4(IV) and α5(IV) subunits (Tryggvason et al, 1993). The laminin molecule is also a heterotrimer of different subunits (Engvall et al, 1990). In classical laminin, the subunits were originally designated A, B1 and B2, but have recently been renamed α1, β1 and γ1 respectively (Burgeson et al, 1994). Subunits M and S, which were described in novel isoforms of laminin, have been renamed α2 and β2 respectively. All isoforms of laminin appear to contain one α chain, one β chain and one γ chain. To avoid confusion with the collagen IV subunits, the laminin subunit names will be given the suffix '(lam)' in this article.

In a recent in situ hybridization study by Pyke et al (1994), mRNA for the novel γ2(lam) subunit was found to be specifically overexpressed by actively invading neoplastic cells in both colorectal and breast carcinomas. This finding may have prognostic significance and underlines the need for detailed studies on the expression of different laminin and collagen IV subunits in cancer. Studying the expression of these subunits in basement membranes around normal structures, such as blood vessels and nerves, may also offer clues to understanding the invasive behaviour of cancers, as there is accumulating evidence that basement membranes regulate the differentiation and behaviour of adjacent cells (Klein et al, 1988; Van Den Hooff, 1989; Sorokin et al, 1990).

We have therefore carried out a descriptive study on normal and neoplastic tissues of colorectum and breast to document for the first time the distribution of the following subunits of collagen IV: α1(IV), α3(IV), α4(IV), α5(IV); and laminin: α1(lam), α2(lam), β1(lam), β2(lam) and γ1(lam).

### MATERIALS AND METHODS

### Antibodies

Anti-collagen IV antibodies used were rabbit polyclonal antiserum raised against human placental collagen-IV and monoclonal clone 1042 (Euro-Path Bude, UK), monoclonal CIV22 (Dako, Bucks, UK) and monoclonal antibodies MAb A10, MAb A2, MAb 85 and MAb A7 (gifts from Dr AF Michael). The polyclonal and monoclonals, clone 1042 and CIV22, are non-subunit specific. MAb A7 recognizes the α5(IV) subunit, which is the same molecule as the Alport antigen (Ding et al, 1994). Monoclonals to the α2(IV) subunit have been difficult to produce, and none were available

*Received 8 November 1995*
*Revised 9 July 1996*
*Accepted 23 July 1996*

*Correspondence to:* RE Hewitt, Laboratory of Pathology, NCI, NIH, Building 10, Room B1B58, 10 CENTER DR MSC 1500, Bethesda MD 20892–1500, USA

**Table 1** Antibodies used in this study

| Antigen | Antibody | Reference |
|---|---|---|
| Laminin | PcAb | |
| α1(lam) | 4C7 | Sanes et al (1990) |
| α2(lam) | 5H2 | Sanes et al (1990) |
| β1(lam) | 4E10 | Sanes et al (1990) |
| β2(lam) | C4 | Sanes et al (1990) |
| γ1(lam) | 2E8 | Sanes et al (1990) |
| Collagen IV | PcAb | |
| Collagen IV | Clone 1042 | Havenith et al (1987) |
| Collagen IV | CIV 22 | Odermatt et al (1984) |
| α1 (IV) | MAb A10 | Ding et al (1994) |
| α3 (IV) | MAb A2 | Ding et al (1994) |
| α4 (IV) | MAb 85 | Ding et al (1994) |
| α5 (IV) | MAb A7 | Ding et al (1994) |

Monoclonal antibody clone designations are given. PcAb, polyclonal antibody.

to us. Anti-laminin antibodies employed were rabbit polyclonal antiserum raised against laminin from mouse EHS sarcoma (Euro-Path), monoclonals 4C7 and 5H2 (Telios Pharmaceuticals, San Diego, CA, USA), monoclonals 4E10 and 2E8 (Chemicon International, Temencula, CA, USA) and monoclonal C4 (a gift from Dr J R Sanes). Antibody specificities and references are given in Table 1. Secondary antibodies included horseradish peroxidase-conjugated swine anti-rabbit and rabbit anti-mouse antibodies (Dako), diluted 1:50 and 1:100 respectively.

### Tissue processing and immunocytochemistry

Samples of normal and neoplastic human colorectal tissue were obtained from bowel specimens received by the Department of Histopathology, Queen's Medical Centre, Nottingham. These included five samples of normal mucosa, five adenomas (four tubular, one villous) and ten moderately differentiated adenocarcinomas. Samples of breast tissue were obtained from specimens received by the Department of Histopathology, City Hospital, Nottingham. All the breast carcinomas studied had been entered into the Nottingham Tenovus Breast Cancer study. Tumour type was classified according to accepted criteria (Ellis et al, 1992). The samples included normal breast tissue in five cases, fibroadenoma in two cases and eight cases of breast carcinoma (three cases of carcinoma of no special type, one tubular carcinoma, one tubular mixed carcinoma, one atypical medullary carcinoma and two lobular carcinomas). Ductal carcinoma in situ (DCIS) was present in five sections of carcinoma, and lobular carcinoma in situ (LCIS) was present in one case. To obtain sections from healing wounds, tissue samples were taken from three mastectomy specimens in which lumpectomy had been performed 23–25 days previously. Samples of normal human kidney were obtained at post mortem.

All tissue samples were quenched in isopentane precooled in liquid nitrogen and stored at –70°C until required for sectioning. Cryostat sections of 4–5 μm were cut, air dried and then fixed in 95% ethanol for 5 min at 4°C. Those sections to be stained with MAb85 and MAb A7 specifically were then denatured (presumed to expose buried epitopes) by incubation in 6M urea, 0.1M glycine HCl, pH 3.5 for 1 h at 4°C (Yoshioka et al, 1985). All sections were then rinsed three times with phosphate-buffered saline (PBS) at room temperature, before staining using a two-step indirect immuno-peroxidase method as described previously (Hewitt et al, 1992).

Sections of normal human kidney were used as a tissue control to demonstrate the reliability of antibody preparations. To test the specificity of staining reactions, sections of colorectal and breast tissue were stained, but with the omission of the primary antibody.

### RESULTS

### Control sections of human kidney

Glomerular BM staining was seen for all antibodies, except those specific for α1(IV), β1(lam) and α2(lam), which instead showed prominent mesangial matrix staining (Figure 1). Both proximal and distal tubular BMs were stained by all antibodies against α1(lam), β1(lam) and γ1(lam), and by anti-α1(IV). Only the distal tubular BMs stained for α3(IV) and α4(IV), and no other novel laminin and collagen-IV subunits were detected in tubular BMs. These results are generally consistent with previous reports (Kleppel et al, 1989; Sanes et al, 1990) and confirm that all antibodies used in this study were working effectively for immunocytochemistry.

### EBM

In normal and neoplastic colorectal tissues (see Table 2 and Figure 2), α1(lam), β1(lam) and γ1(lam) were detected in the EBMs, as was α1(IV). EBM staining was generally continuous in normal mucosa and adenomas, but discontinuous in all the carcinomas observed (Figure 3). There was little evidence of novel subunit expression [i.e. subunits α3(IV), α4 (IV), α5(IV), α2(lam) or β2(lam)] in EBMs of either normal or neoplastic colorectal tissues. The only examples were (1) weak staining for α3(IV) subunit beneath the surface epithelium of normal mucosa; (2) weak staining for α2(lam) at the bases of glands in normal mucosa; and (3) focal α2(lam) staining in adenomas.

**Table 2** Immunostaining of EBMs in normal and neoplastic tissues of colorectum and breast

| | Colorectal | | | Breast | | | | |
|---|---|---|---|---|---|---|---|---|
| Antigen | NM | A | C | N | F | DCIS | NST | L |
| Laminin (PcAb) | 2+ | 2+ | –/3+ | 2+ | + | 2+ | –/+ | –/+ |
| α1(lam) | 2+ | 2+ | –/3+ | 3+ | 3+ | 3+ | –/2+ | – |
| α2(lam) | (+)[b] | (2+)[f] | – | – | + | + | – | – |
| β1(lam) | 3+ | 2+ | –/3+ | 3+ | 3+ | 3+ | –/2+ | –/+ |
| β2(lam) | – | (+)[f] | – | 2+ | 2+ | 2+ | – | – |
| γ1(lam) | 3+ | 2+ | –/2+ | 3+ | 3+ | 3+ | –/2+ | –/+ |
| Collagen IV (PcAb) | 3+ | 3+ | –/3+ | 3+ | 3+ | 3+ | –/2+ | –/+ |
| Collagen IV (clone 1042) | 2+ | 2+ | –/3+ | 3+ | 2+ | 3+ | –/2+ | – |
| Collagen IV (CIV 22) | 2+ | 2+ | –/3+ | 3+ | 2+ | 2+ | –/+ | – |
| α1 (IV) | + | + | –/+ | 2+ | 2+ | + | –/+ | – |
| α3 (IV) | (+)[s] | – | – | – | – | – | – | – |
| α4 (IV) | – | – | – | – | – | – | – | – |
| α5 (IV) | – | – | – | – | + | – | – | – |

Not all cases of breast carcinoma are represented, but scores for tubular and atypical medullary carcinomas followed the same pattern as shown for the NST carcinomas. The tubular mixed carcinomas only showed weak staining with the collagen-IV polyclonal and monoclonals, 1042 and CIV22. The LCIS showed a similar pattern to the cases of DCIS. Scoring of staining intensity: 3+, strong; 2+, moderate; +, weak; – absent; –/3+, strong but discontinuous. NM, normal colorectal mucosa; A, colorectal adenoma; C, colorectal carcinoma; N, normal breast tissue; F, fibroadenoma; NST, carcinomas of no special type; L, lobular carcinoma. [b] gland base;[s] surface epithelium;[f], focal.

© Cancer Research Campaign 1997

**Figure 1** Kidney control sections stained for various antibodies to collagen IV. Staining with polyclonal anti-collagen IV (**A**) detects antigen in the glomerular BM (short arrow), Bowman's capsule (long arrow) and the renal tubule BM (arrowheads). The monoclonal clone 1042 (**B**) gave a similar pattern. The anti-$\alpha$1(IV) monoclonal (**C**) also stained Bowman's capsule and the tubular BM, but stained mesangial matrix (arrowheads) rather than glomerular BM. Antibodies against $\alpha$3(IV) and $\alpha$4(IV) subunits, (**D**) and (**E**) respectively, stained the glomerular BM, Bowman's capsule and BMs of the distal (arrow) but not proximal tubules. Staining for the $\alpha$5(IV) subunit (**F**) was only seen in the glomerular BM and Bowman's capsule

In normal breast tissue (see Table 2), there was EBM staining for $\alpha$1(lam), $\beta$1(lam), $\gamma$1(lam) and $\alpha$1(IV), as well as for $\beta$2(lam) unlike colorectal tissues. In fibroadenomas and in situ carcinoma, the EBM stained additionally for $\alpha$2(lam), and fibroadenomas also showed weak EBM staining for the $\alpha$5(IV) subunit. Where EBM staining was present in these lesions, it was continuous, with the exception of the strong but patchy staining for $\alpha$2(lam) in the lobular carcinoma in situ (LCIS). By contrast, invasive breast carcinomas invariably showed either discontinuous or absent EBM staining. Discontinuous staining was seen for $\alpha$1(lam), $\beta$1(lam) and $\gamma$1(lam), and for $\alpha$1(IV). No EBM staining for $\alpha$2(lam), $\beta$2(lam), $\alpha$3(IV), $\alpha$4(IV) or $\alpha$5(IV) was seen in any invasive breast carcinoma, with the exception of one atypical medullary carcinoma, which showed weak patchy staining for the $\beta$2(lam).

### Blood vessels, lymphatics and nerves

The capillaries of normal colorectal mucosa and adenomas stained for $\alpha$1(lam), $\beta$1(lam), $\beta$2(lam), $\gamma$1(lam) and $\alpha$1(IV). The capillaries of normal breast tissue and fibroadenomas also stained for these antigens and, in addition, stained for $\alpha$2(lam) and $\alpha$5(IV). Capillaries of both colorectal and breast carcinomas gave similar staining results to

© Cancer Research Campaign 1997

**Figure 2** Immunostaining of normal human colorectal tissues. The clone 1042 anti-type IV collagen monoclonal stains a variety of structures in normal mucosa (**A**), including the EBM (large arrow) and cells with a dendritic-type morphology (small arrows). Staining for the α3(IV) subunit (**B**) is restricted to the EBM beneath the luminal surface epithelium (large arrow) and non-specifically stained macrophages (small arrows). Staining for the Alport antigen (**C**) is seen in nerves (arrow), but not blood vessels of the submucosa (arrowhead). In (**D**), staining for β2(lam) is seen in both nerves (arrow) and blood vessels (arrowhead) of the submucosa, but not large lymphatic vessels (ly). Staining patterns for γ1(lam) (**E**) and β1(lam) (**F**) differ in that arterial smooth muscle only stains for γ1(lam) (black arrowhead). Staining for both antigens is seen in the BM surrounding ganglia of the myenteric plexus (white arrows) and around individual smooth muscle cells of the muscularis propria corresponding normal tissues, with the exception that staining for β2(lam) was weak or absent in carcinomas (see Table 3 and Figure 4). Sections taken from three different healing wounds (23–25 days old) were also immunostained. In areas of wound healing, the capillaries showed weak β2(lam) staining, but staining was stronger in capillaries of the adjacent normal tissue (Figure 4).

Larger blood vessels, seen most often in normal colorectal tissues, showed staining for α1(lam), β1(lam), β2(lam), γ1(lam) and α1(IV). One prominent feature was that in arteries, the smooth muscle layer showed a selective absence of staining for the β1(lam) subunit (Figure 2F).

Larger lymphatic channels in the colorectal submucosa stained for α1(IV), β1(lam) and γ1(lam), but unlike blood vessels they did not stain for α1(lam) or β2(lam) (Figure 2 D). Lymphatic capillaries were not examined here, as these vessels were too difficult to identify in our cryostat sections.

Autonomic nerves and ganglia seen in colorectal tissues stained with all antibodies used in this study, except antibodies to the α3(IV) and α4(IV) subunits. Unlike any other component of colorectal tissues, autonomic nerves and ganglia did stain for the α5(IV) subunit.

© Cancer Research Campaign 1997

**Figure 3** Immunostaining of sections of a colonic carcinoma with polyclonal anti-laminin antibody. In the photomicrograph (**A**), continuous staining of the EBM is seen within the tumour (arrows), but no staining is seen at the invasive edge (arrowheads). These features are emphasized in the schematic diagram (**B**), in which connective tissue is represented in white, neoplastic cell islands and glands in grey, and basement membranes that envelop the neoplastic cell areas are shown in black

### Smooth muscle and stromal myofibroblasts

Smooth muscle cells of muscularis mucosae and propria in the colorectum showed moderate or strong staining for all antigens studied (Figure 2), with the exception of α2(lam), α3(IV), α4(IV) and α5(IV), for which there was no staining.

Cells with dendritic-type morphology having multiple long thin processes were also observed between the glands in normal colorectal mucosa (Figure 2A). These cells were most obvious in sections stained with antibody to α2(lam), as this did not stain neighbouring structures such as EBM, blood vessels or muscular layers of the bowel wall. Preliminary ultrastructural studies suggest that these unusual cells with multiple processes are myofibroblast-like (T Gray and RE Hewitt, unpublished observations).

In both colorectal and breast carcinomas, stromal myofibroblasts showed weak or absent staining for α1(lam) (see Figure 5), despite moderately strong staining for β1(lam) and γ1(lam) subunits and for α1(IV). In sections stained for α1(lam), the EBMs are therefore especially prominent.

### DISCUSSION

There have been many immunocytochemical studies on basement membrane staining in colorectal and breast neoplasms. However, with only a very few exceptions, the anti-laminin and anti-collagen IV antibodies used in such studies have been non-subunit specific. Now that subunit-specific antibodies and DNA probes are available, it is possible to obtain more detailed and meaningful information about the interactions between tumour cells, their EBMs and other basement membranes they encounter. For example, in a previous in situ hybridization study using a cDNA probe for α1(IV) (Hewitt et al, 1992), we found that the hybridization signal was entirely localized to blood vessels. This raised the possibility that the collagen IV demonstrated in the EBMs by immunocytochemistry might consist of novel collagen IV isoforms. However, in the light of our present results this seems less likely, as the EBMs of colorectal carcinomas stained for the α1(IV) subunit of classical collagen IV, but not for α3(IV), α4(IV) and α5(IV).

**Table 3** Capillary immunostaining in normal and neoplastic tissues of colorectum and breast

| Antigen | Colorectal NM | Colorectal A | Colorectal C | Breast N | Breast F | Breast C |
|---|---|---|---|---|---|---|
| Laminin (PcAb) | 2+ | 3+ | 3+ | + | + | 2+ |
| α1(lam) | 2+ | 3+ | 2+ | 3+ | 3+ | 3+ |
| α2(lam) | – | – | – | + | + | – |
| β1(lam) | 2+ | 3+ | 3+ | 3+ | 3+ | 3+ |
| β2(lam) | + | 2+ | –/+ | 3+ | 2+ | + |
| γ1(lam) | 3+ | 3+ | 3+ | 3+ | 3+ | 3+ |
| Collagen IV (PcAb) | 3+ | 3+ | 3+ | 2+ | 2+ | 3+ |
| Collagen IV (clone 1042) | 3+ | 3+ | 3+ | 3+ | 2+ | 3+ |
| Collagen IV (CIV 22) | 3+ | 3+ | 3+ | 2+ | 2+ | 2+ |
| α1 (IV) | 2+ | 2+ | 2+ | 2+ | 2+ | 2+ |
| α3 (IV) | – | – | – | – | – | – |
| α4 (IV) | – | – | – | – | – | – |
| α5 (IV) | – | – | – | + | 2+ | – |

NM, normal mucosa; N, normal breast tissue; A, adenoma; F, fibroadenoma; C, carcinoma.

Pyke et al (1994) have recently reported an in situ hybridization study using probes for α1(lam), β1(lam) and γ1(lam) to analyse colonic cancers. In the 16 colon carcinomas studied, the hybridization signal for these three probes was weak and was consistently localized both to stromal cells with fibroblast-like morphology and to the endothelial cells of small vessels. These findings are consistent with the results of the present study, in that α1(lam), β1(lam) and γ1(lam) were all detected in blood vessel walls by immunocytochemistry. Presumably, endothelial cells contribute to the synthesis of laminin deposited in the blood vessel wall. Pyke et al (1994) never found expression of α1(lam), β1(lam) and γ1(lam) in neoplastic cells of colon cancers, which may indicate that EBM laminin in these tumours is derived mainly or entirely from stromal fibroblasts. As mentioned previously, Pyke et al (1994) did find a marked increase in γ2(lam) expression in neoplastic cells at the invasive edge of colorectal cancers. We did not find evidence for any similar increases in expression of laminin or collagen-IV subunits, although γ2(lam) was not examined in this study.

### EBM staining patterns

In non-malignant tissues of both colorectum and breast tissue, we have found obvious EBM staining for subunits of classical laminin [α1(lam), β1(lam) and γ1(lam)] and for the α1(IV) subunit of classical collagen IV. Staining for all these antigens was patchy in

© Cancer Research Campaign 1997

**Figure 4** Vascular staining patterns in carcinomas and in healing wounds. Widespread vascular staining (arrows) was seen in sections of colonic carcinoma (**A**), tubular mixed breast carcinoma (**C**) and a 23-day-old healing breast wound (**E**), when stained with antibodies against classical collagen-IV and laminin subunits [c1042 monoclonal, anti-$\alpha$1(lam) and anti-$\gamma$1(lam) respectively]. However, with antibody to $\beta$2(lam), there was no vascular staining in either the colonic carcinoma (**B**) or the breast carcinoma (**D**). Similarly, for the breast wound (**F**), there was little evidence of vascular staining for $\beta$2(lam) in the healing wound itself (hw), but there was vascular staining in the adjacent normal tissue carcinomas, which is consistent with the fact that EBMs are discontinuous in these tumours. The myofibroblasts in breast and colorectal carcinomas showed staining for $\beta$1(lam) and $\gamma$1(lam), but not obviously for $\alpha$1(lam). Consequently, staining with antibody to $\alpha$1(lam) demonstrated the EBMs most specifically, with the least background stromal staining. Use of antibody to $\alpha$1(lam) should therefore be advantageous for studies on EBM staining patterns in cancer.

In both colorectal and breast tissues, there was a lack of EBM staining for novel $\alpha$3(IV) and $\alpha$4(IV) subunits. Weak staining for the $\alpha$5(IV) subunit was seen in the EBM of breast fibroadenomas, but in no other tissue. For both colorectal and breast tissues, $\alpha$2(lam) showed a sporadic staining pattern. While EBM staining for the antigen was generally weak or absent, intense focal staining was seen in an area of LCIS, and moderately strong focal staining

© Cancer Research Campaign 1997

**Figure 5** Immunostaining of stromal cells in carcinomas. Serial sections of a colonic adenocarcinoma were stained for α1(lam) (**A**) and β1(lam) (**B**). In the same way, serial sections of lobular breast carcinoma were stained for α1(lam) (**C**) and β1(lam) (**D**). For both types of carcinoma, stromal components were stained for β1(lam), but not α1(lam). In the lobular carcinoma there was no evidence of EBM staining, except in an area of in situ carcinoma or LCIS (L)

was seen in colorectal adenomas. The significance of this finding is unclear at present.

An interesting difference was observed between non-malignant tissues of colorectum and breast. While β2(lam) was not detected in the EBM of non-malignant colorectal tissues, it was consistently detected in EBMs of normal breast tissues, including normal breast, fibroadenomas and both ductal and lobular carcinoma in situ. This difference in the composition of EBM in normal tissues of breast and colorectum may be the result of different synthetic capabilities of epithelial cells at these two sites. Equally, it may influence the type of differentiation shown by epithelia in these two locations.

While β2(lam) staining was present in EBMs of non-malignant breast tissues, it was almost completely absent from the EBMs of breast carcinomas. This suggests that in tumours of the breast, levels of β2(lam) staining may be inversely related to invasive activity. It is possible that loss of the subunit facilitates the invasive process, but its loss may also be secondary to invasive activity. In contrast, staining for α2(lam) was not seen in the EBMs of either normal breast tissues or frankly invasive cancers, but was seen in fibroadenomas and in situ carcinomas of the breast. Further studies on the distribution of β2(lam) and α2(lam) in breast neoplasia seem worthwhile, as they may help in the differential diagnosis of benign and malignant lesions.

Other evidence that the β2(lam) content of EBMs deserves further investigation comes from a report by Sollberg et al (1992), who found the EBM of nodular basal cell carcinomas does stain for β2(lam), while the EBM of normal epidermis does not. Taken together, all this evidence suggests that the β2(lam) content of EBMs varies greatly, depending on the character of the adjacent cells.

### Blood vessel staining

Vascular staining patterns in breast and colorectal tissues were similar and showed prominent staining for all the subunits of classical laminin, and for the α1(IV) subunit of classical collagen IV. This abundance of BM proteins may facilitate tumour cell attachment to the walls of blood vessels, which may in turn lead to dissolution of the vessel wall and penetration into the vascular lumen, according to the 'three-step' hypothesis of invasion (Liotta et al, 1983).

© Cancer Research Campaign 1997 

While vascular staining for β2(lam) was prominent in non-malignant tissues of breast and colorectum, it was often weak or absent in the vessels within carcinomas. Interestingly, we also found that the newly formed and poorly differentiated blood vessels of healing wounds showed deficient β2(lam) staining. There is abundant evidence that blood vessels of carcinomas show incomplete differentiation, as they are often dilated with irregular endothelial linings, scanty perivascular connective tissues and tortuous, disorganized courses (Willis, 1973; Denekamp, 1983). Furthermore, in an extensive study on human tumours, Lindgren (1945) found that vascular architecture was most abnormal in the less well-differentiated malignant tumours. It is therefore possible that the low level of vascular staining for β2(lam) in carcinomas is a result of the incomplete differentiation of the tumour blood vessels.

Although vascular staining in colorectal and breast tissues showed generally similar patterns, a difference was seen with respect to staining for the α5(IV) subunit. In colorectal tissues, only the autonomic nerves and ganglia stained for this antigen. However, in normal breast tissue there was weak staining of capillaries and larger blood vessels, and moderately strong vascular staining was seen in fibroadenomas. This suggests a difference in BM composition between blood vessels of normal colorectal and breast tissues.

We previously used an α1(IV) subunit cDNA probe for an in situ hybridization (ISH) study on colorectal carcinomas (Hewitt et al, 1992) and found a high level of mRNA expression in some tumour blood vessels, but no detectable expression in any other tumour component. This pattern of expression seems consistent with the present immunocytochemical results, which suggest that the α1(IV) subunit is more abundant in the walls of tumour blood vessels than in the EBM or any other tumour component. It may be relevant that blood vessels in some cancers produce high levels of the protease inhibitor, plasminogen activator inhibitor 1 (PAI-1), and it has been hypothesized that this is part of a protective response to prevent proteases produced within the tumour from degrading the tumour's own vascular connective tissue stroma (Kristensen et al, 1990). The high levels of collagen IV production in blood vessels of colorectal cancer (Hewitt et al, 1992) may be part of the same protective response.

### The significance of differences in BM composition

The varied laminin and collagen-IV subunit composition of different BMs raises the interesting question of why there should be a biological need for this level of complexity. One reason may be that different subunits impart different physical properties to BMs, such as differences in permeability. For example, in the kidney, the high volume unidirectional transfer of fluid may be facilitated by the high levels of novel collagen IV subunits in the glomerular BMs (Kleppel et al, 1989). Another reason for the complexity may be that different subunits may have important regulatory effects on the biological activities of adjacent cells. Supporting evidence comes from the fact that β2(lam), unlike β1(lam), contains sequences selective for the attachment of motoneurones (Sanes et al, 1990). As β2(lam) is abundant in synaptic BM, this may explain why the regenerating axons in denervated muscle preferentially form neuromuscular junctions at the original synaptic sites (Hunter et al, 1989). β2(lam) therefore appears to have very specific effects on the behaviour of at least one cell type. The selective loss of β2(lam) from epithelial and vascular BMs in cancers, observed in the present study, may therefore have important effects on the behaviour of adjacent neoplastic and endothelial cells.

### CONCLUSION

The findings of this study show that the α2(lam) subunit and the novel chains of collagen-IV [α3 (IV), α4 (IV) and α5(IV)] show limited expression in normal and neoplastic tissues of colorectum and breast. In contrast, the subunits of classical laminin [α1(lam), β1(lam) and γ1(lam)] and the α1(IV) chain are very widely distributed in the basement membranes of these tissues. To our knowledge, this is the first immunocytochemical study to document the distribution of any of the subunits of laminin and collagen IV in either colorectal or breast cancer. It is hoped that the information presented here will provide a useful reference point for future investigations.

### ACKNOWLEDGEMENTS

We are grateful to Mr William Brackenbury for his skilled photographic assistance, to Ms Jane Bell, Mrs Helen Naylor and Mr Peter Wencyk for technical assistance. We would also like to thank Drs AF Michael, MM Kleppel and JR Sanes for providing monoclonal antibodies as gifts. This work was supported by a grant from the Special Trustees of the Nottingham Hospitals and a generous donation from Dr M Busson of Boots Pharmaceuticals.

#### REFERENCES

Albrechtsen R, Nielsen M, Wewer U, Engvall E and Ruoslahti E (1981) Basement membrane changes in breast cancer detected by immunohistochemical staining for laminin. *Cancer Res* **41**: 5076–5081

Barsky SH, Siegal GP, Jannotta F and Liotta LA (1983) Loss of basement membrane components by invasive tumours but not by their benign counterparts. *Lab Invest* **49**: 140–147

Bosman FT (1994) The borderline: basement membranes and the transition from premalignant to malignant neoplasia. *Microsc Res Tech* **28**: 216–225

Burgeson RE, Chiquet M, Deutzmann R, Ekblom P, Engel J, Kleinman H, Martin GR, Meneguzzi G, Paulsson M, Sanes J, Timpl R, Tryggvason K, Yamada Y and Yurchenco PD (1994) A new nomenclature for laminins. *Matrix Biol* **14**: 209–211

D'Ardenne AJ (1989) Use of basement membrane markers in tumour diagnosis: a review. *J Clin Pathol* **42**: 449–457

Denekamp J, Hill SA and Hobson B (1983) Vascular occlusion and tumour cell death. *Eur J Cancer Clin Oncol* **19**: 271–275

Ding J, Kashtan CE, Fan WW, Kleppel MM, Sun MJ, Kalluri R, Neilson EG and Michael AF (1994) A monoclonal antibody marker for Alport syndrome identifies the Alport antigen as the alpha 5 chain of type IV collagen. *Kidney Int* **45**: 1504–1506

Ellis IO, Galea M, Broughton N, Locker A, Blamey RW and Elston CW (1992) Pathological prognostic factors in breast cancer. II. Histological type. Relationship with survival in a large study with long-term follow-up. *Histopathology* **20**: 479–489

Engvall E, Earwicker D, Haaperanta T, Ruoslahti E and Sanes JR (1990) Distribution and isolation of four laminin variants; tissue restricted distribution of heterotrimers assembled from five different subunits. *Cell Reg* **1**: 731–740

Forster SJ, Talbot IC, Clayton DG and Critchley DR (1986) Tumour basement membrane laminin in adenocarcinoma of rectum: an immunohistochemical study of biological and clinical significance. *Int J Cancer* **37**: 813–817

Havenith MG, Arends JW, Simon R, Volovics A, Wiggers T and Bosman FT (1988) Type IV collagen immunoreactivity in colorectal cancer. *Cancer* **62**: 2207–2211

Havenith MG, Cleutjens JPM, Beek C, van der Linden E, De. Goeij AFPM and Bosman FT (1987) Human specific anti-type IV collagen monoclonal antibodies, characterization and immunohistochemical application. *Histochemistry* **87**: 123–128

© Cancer Research Campaign 1997

Hewitt RE, Powe DG, Griffin NR and Turner DR (1991) Relationships between epithelial basement membrane staining patterns in primary colorectal carcinomas and the extent of tumour spread. *Int J Cancer* **48**: 855–860

Hewitt RE, Powe DG, Carter GI, Turner DR and Price JE (1992) Basement membrane collagen-IV synthesis in colorectal tumours. *Int J Cancer* **51**: 530–536

Hunter DD, Porter BE, Bulock JW, Adams SP, Merlie JP and Sanes JR (1989) Primary sequenc of a motor neurone-selective adhesive site in the synaptic basal lamina protein s-laminin. *Cell* **59**: 905–913

Klein G, Langegger M, Timpl R and Ekblom P (1988) Role of laminin A chain in the development of epithelial cell polarity. *Cell* **55**: 331–341

Kleppel MM, Santi PA, Cameron JD, Wieslander J and Michael AF (1989) Human tissue distribution of novel basement membrane collagen. *Am J Pathol* **134**: 813–825

Kristensen P, Pyke C, Lund LR, Andreasen PA and Dano K (1990) Plasminogen activator inhibitor type-1 in Lewis lung carcinoma. *Histochemistry* **93**: 559–566

Lindgren AGH (1945) The vascular supply of tumours with special reference to the capillary angioarchitecture. *Acta Pathol Microbiol Scand* **22**: 493–521

Liotta LA, Rao CN and Barsky S (1983) Tumour invasion and the extracellular matrix. *Lab Invest* **49**: 636–649

Odermatt BF, Land AB, Rüttner JR, Winterhalter KH and Trüeb B (1984) Monoclonal antibodies to human type IV collagen: useful reagents to demonstrate the heterotrimeric nature of the molecule. *Proc Natl Acad Sci USA* **81**: 7343–7347

Pyke C, Romer J, Kallunki P, Lund LR, Ralfkiaer E, Dano K and Tryggvason K (1994) The gamma 2 chain of kalinin/laminin 5 is preferentially expressed in invading malignant cells in human cancers. *Am J Pathol* **145**: 782–791

Sanes JR, Engvall E, Butkowski R and Hunter DD (1990) Molecular heterogeneity of basal laminae: isoforms of laminin and collagen IV at the neuromuscular junction and elsewhere. *J Cell Biol* **111**: 1685–1699

Sollberg S, Peltonen J and Uitto J (1992) Differential expression of laminin isoforms and β4 integrin epitopes in the basement membrane zone of normal human skin and basal cell carcinomas. *J Invest Dermatol* **98**: 864–870

Sorokin L, Sonnenberg A, Aumailley M, Timpl R and Ekblom P (1990) Recognition of the laminin-E8 cell-binding site by an integrin possessing the alpha-6 subunit is essential for epithelial polarization in developing kidney tubules. *J Cell Biol* **111**: 1265–1273

Tryggvason K, Zhou J, Hostokka SL and Shows TB (1993) Molecular genetics of Alport syndrome. *Kidney Int* **43**: 38–44

Willis RA (1973) *Spread of Tumours in the Human Body*. Butterworths: London

Yoshioka K, Michael AF, Velosa J and Fish AJ (1985) Detection of hidden nephritogenic antigen determinants in human renal and non-renal basement membranes. *Am J Pathol* **121**: 156–165

Van Den Hooff A (1989) An essay on basement membranes and their involvement in cancer. *Persp Biol Med* **32**: 401–413

© Cancer Research Campaign 1997